# Average of Cases of Consumption Cured

**Published:** 1845-10

**Authors:** 


*Average of cases of Consumption Cured.*—M. Roudet states, that in the post-mortem examinations of forty-five subjects, between three and fifteen years old, he had observed the cure of consumption in twelve cases. In one hundred and sixteen individuals, aged between fifteen and seventy-six years, tubercles in the lung or bronchial glands had become innocuous in ninety-seven cases, and had wholly disappeared in sixty-one. In one hundred and ninety-seven autopsies promiscuously taken, he found ten instances in which, at least, one cavern completely cicatrized, existed in the lung; and in eight cases one or more cavities were found in different stages of cicatrization.—*Ibid.*

